# TLR9-ERK-mTOR signaling is critical for autophagic cell death induced by CpG oligodeoxynucleotide 107 combined with irradiation in glioma cells

**DOI:** 10.1038/srep27104

**Published:** 2016-06-02

**Authors:** Xiaoli Li, Yanyan Cen, Yongqing Cai, Tao Liu, Huan Liu, Guanqun Cao, Dan Liu, Bin Li, Wei Peng, Jintao Zou, Xueli Pang, Jiang Zheng, Hong Zhou

**Affiliations:** 1 Department of Pharmacology, College of Pharmacy, the Third Military Medical University, Chongqing 400038, China; 2 Department of Pharmacy, Institute of Surgery Research, Daping Hospital, the Third Military Medical University, Chongqing 400042, China; 3 Company 18th, College of Pharmacy, the Third Military Medical University, Chongqing 400038, China; 4 Department of Oncology, Southwest Hospital, the Third Military Medical University, Chongqing 400038, China; 5 Medical Research Center, Southwestern Hospital, the Third Military Medical University, Chongqing 400038, China

## Abstract

Synthetic oligodeoxynucleotides containing unmethylated CpG dinucleotides (CpG ODN) function as potential radiosensitizers for glioma treatment, although the underlying mechanism is unclear. It was observed that CpG ODN107, when combined with irradiation, did not induce apoptosis. Herein, the effect of CpG ODN107 + irradiation on autophagy and the related signaling pathways was investigated. *In vitro*, CpG ODN107 + irradiation induced autophagosome formation, increased the ratio of LC3 II/LC3 I, beclin 1 and decreased p62 expression in U87 cells. Meanwhile, CpG ODN107 also increased LC3 II/LC3 I expression in U251 and CHG-5 cells. *In vivo*, CpG ODN107 combined with local radiotherapy induced autophagosome formation in orthotopic transplantation tumor. Investigation of the molecular mechanisms demonstrated that CpG ODN107 + irradiation increased the levels of TLR9 and p-ERK, and decreased the level of p-mTOR in glioma cells. Further, TLR9-specific siRNA could affect the expressions of p-ERK and autophagy-related proteins in glioma cells. Taken together, CpG ODN107 combined with irradiation could induce autophagic cell death, and this effect was closely related to the TLR9-ERK-mTOR signaling pathway in glioma cells, providing new insights into the investigation mechanism of CpG ODN.

Surgery combined with radiotherapy is a standard treatment for patients with glioma; however, the therapeutic outcome is poor because tumor cells often become radioresistant^1^,^2^. Increased radiation dosage can improve local control of tumor development but could induce serious side effects^3^,^4^. Therefore, the identification of radiosensitizers to increase the sensitivity of glioma to normal radiotherapy regimens is very important.

Glioma commonly develops resistance to cell death either by disruption of apoptotic processes or by activation of survival signals^5^. Therefore, it is essential to investigate the agents capable of inhibiting tumor cell proliferation or inducing tumor cell death. To date, several studies have considered CpG oligodeoxynucleotides (CpG ODN), synthetic oligodeoxynucleotides (ODN) containing unmethylated CpG motifs, as a promising type of radiosensitizer^6^,^7^. We also observed its strong radiosensitizing effect against glioma cells of CpG ODN107 (5′-TGGCGCGGGCGG-3′), newly identified in our lab, which promoted cell death and inhibited cell growth both *in vitro* and *in vivo*^8^,^9^.

Recently, autophagy has been proven to be an important component of many critical biological processes^10^,^11^. Moreover, it is closely related to human diseases such as cancer and is an important factor in successful tumor therapy^12^,^13^,^14^. However, the initiation signal for autophagy is poorly understood, although several molecules and signaling pathways have been investigated. The kinase mTOR is a critical regulator of autophagy, which is activated through the phosphatidylinositol 3-kinase/PKB (PI3K-Akt) and mitogen-activated protein kinases (MAPKs) signaling pathways to suppress autophagy. AMP-activated protein kinase (AMPK) and phosphatase and tensin homolog (PTEN) signaling negatively regulate mTOR signaling to promote autophagy^15^,^16^.

Recent evidence has suggested that autophagy is involved in the mechanism of cell death in irradiated glioma cells^17^, and that CpG ODN could induce autophagy in rodent and human tumor cell lines in a TLR9-dependent manner^18^. Based on our previous finding that CpG ODN107+ irradiation did not induce apoptosis^8^, we speculated the existence of at least one another mode of death. Thus, in this study, we examined the effect of CpG ODN107+ irradiation on autophagy and investigated the possible signaling pathway involved in autophagy. Our results showed that CpG ODN107+ irradiation induced autophagy both *in vitro* and *in vivo*. The mechanism underlying the radiosensitizing effect of CpG ODN107 was closely related to autophagy, with the TLR9-ERK-mTOR signaling pathway being critical for the induction of autophagic cell death in glioma cells.

## Results

### CpG ODN107+ irradiation significantly increases autophagy and inhibition of autophagy leads to loss of radiosensitizing effect of CpG ODN107 *in vitro*

#### CpG ODN107, irradiation, and CpG ODN107+ irradiation induce autophagosome formation in U87 cells

Previously, our results demonstrated that U87 cells are insensitive to 5 Gy of irradiation; however, 10 μg/mL of CpG ODN107 combined with 5 Gy of irradiation could significantly inhibit the proliferation and viability of U87 cells^8^,^9^. In this study, after investigating the effects of different irradiation doses on autophagosome formation and autophagy-related protein expression (Figs S1 and S2), a dose of 5 Gy of irradiation, insensitive for glioma cells, and 10 μg/mL of CpG ODN107 was selected and used in our experiments.

**Figure 1:**
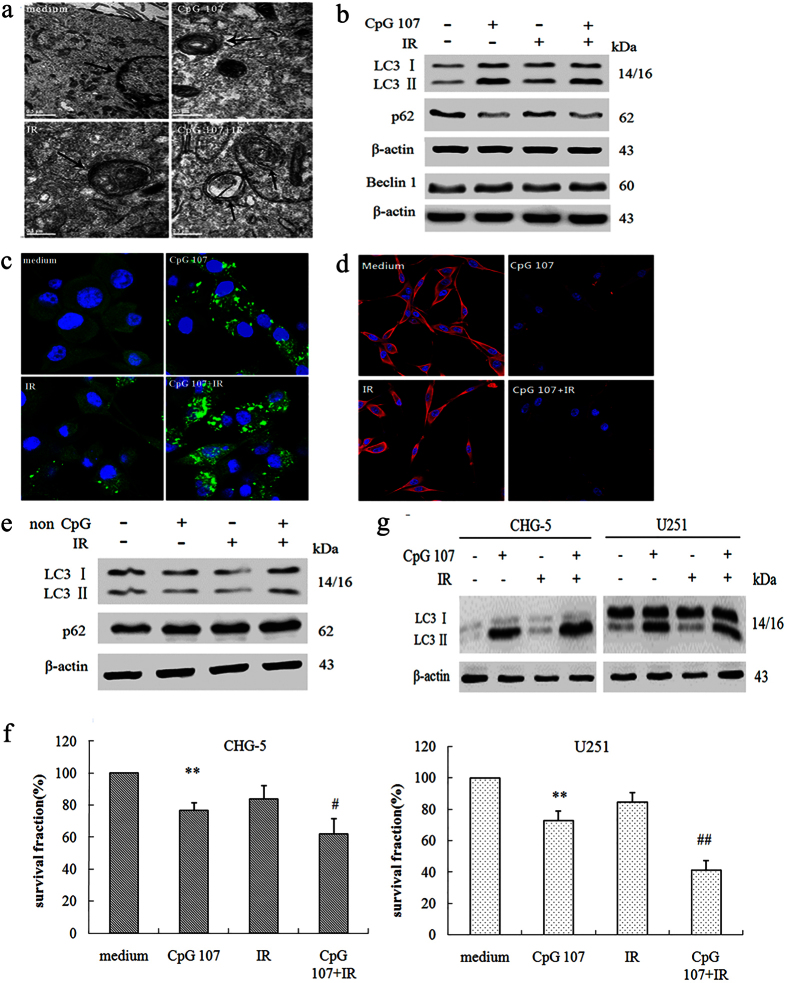
CpG ODN107+ irradiation induces autophagy in glioma cells. (**a**) Autophagosomes observed by transmission electron microscopy (TEM) in U87 cells. Cells were treated with 10 μg/mL of CpG ODN107 for 12 h, and then treated with or without 5 Gy of irradiation. After incubation for 24 h, TEM observation was carried out. Autophagosomes (marked by arrows) were presented as rounded vacuolar structures with double or multiple membranes surrounding the cytoplasmic contents. (**b**) LC3, p62 and beclin 1 proteins expressions by western blotting assay in U87 cell. The cells were treated as described as (**a**), except the cells were collected for western blotting assay. (**c**) GFP-LC3 fluorescence images and quantitative analysis are shown. U87 cells with stable GFP-LC3 expression were treated as described as (**a**), except the cells were collected for confocal immunofluorescent assays. Autophagosome is shown as green punctate structure in U87/GFP-LC3. ^##^*P *< 0.01 vs. Medium; **P < 0.01 vs. IR. Error bars represent the mean ± S.D. (**d**) p62 protein expression observed by confocal immunofluorescent analysis in U87 cells (×400). Cells were treated as described as (**a**), except the cells were collected for confocal immunofluorescent assays. Red fluorescence represents p62 protein. (**e**) LC3 and p62 proteins expressions by western blotting assay in U87 cells exposed to non-CpG ODN combined with irradiation. Cells were treated with 10 μg/mL of non-CpG ODN for 12 h, and then treated with or without 5 Gy of irradiation. After incubation for 24 h, the cells were collected for western blotting assay. (**f**) The proliferation of U251 and CHG-5 cells exposed to CpG ODN107 combined with irradiation. Cells were treated as described as (**a**), except the cells were collected for colony formation assay. The survival fraction (%) was calculated according to the following formula: colony number of treated group/colony number of medium group ×100%. Error bars represent the mean ± S.D. (n = 3). **P < 0.01 vs. medium; ^#^*P* < 0.05 or ^##^*P* < 0.01 vs. IR. (**g**) LC3 proteins expressions by western blotting assay in U251 and CHG-5 cells. Cells were treated as described as (**a**), except the cells were collected for western blotting assay. In figures, CpG ODN107 was abbreviated as CpG 107; non-CpG ODN was abbreviated as non-CpG; irradiation was abbreviated as IR.

Transmission electron microscopy (TEM), the gold standard method for determination of autophagy^19^, was used to confirm the induction of autophagy in U87 cells by CpG ODN107+ irradiation. Rounded vacuolar structures with double or multiple membranes surrounding the cytoplasmic contents were observed within U87 cells treated with irradiation alone, CpG ODN107 alone or CpG ODN107+irradiation. However, no such structure was observed within the U87 cells in the medium group (Fig. 1a).

#### CpG ODN107+ irradiation significantly increases autophagy-related protein expression in U87 cells

Beclin 1, LC3 (light chain 3) and p62 are essential for autophagy and were selected as autophagy-related proteins in this study. LC3 is distributed in the autophagosome membrane^20^. The LC3 protein expression, especially the protein expression ratio of LC3 II to LC3 I (LC3 II/LC3 I), is considered as an accurate indicator of autophagosome formation^21^,^22^. In this study, the results of western blotting showed that the ratio of LC3 II/LC3 I increased after CpG ODN107+ irradiation treatment compared with that when irradiation alone was used (Fig. 1b). Further, the formation of punctate GFP-LC3, the marker of autophagy, in U87 cells with stable GFP-LC3 expression was observed using laser confocal scanning. The results showed green fluorescence diffused throughout the cells without any treatment (medium). However, the green fluorescence presented as punctate in the cells treated with CpG ODN107+ irradiation, and the number of cells (nearly 78%) containing punctate GFP-LC3 was significantly greater compared with the cells treated with irradiation alone, suggesting that CpG ODN107+ irradiation significantly increased autophagosome formation (Fig. 1c).

Beclin 1 and p62 proteins are other indicators of autophagosome formation^20^,^23^. The results from western blotting assay showed beclin 1 expression obviously increased following treatment of CpG ODN107+ irradiation compared with that of irradiation alone (Fig. 1b). Meanwhile, the results of immunofluorescence assay (red fluorescence represents p62 protein) and western blotting showed that p62 expression within the cells treated with CpG ODN107 obviously decreased compared with that in the cells without any treatment, and also decreased within the cells treated with CpG ODN107+ irradiation (Fig. 1b,d). The results from the two assays were consistent.

Previous results showed that a non-CpG ODN had no radiosensitizing effect against glioma cells^8^. In this study, the influence of non-CpG ODN as a negative control was observed on autophagy as well. The results showed that non-CpG ODN+ irradiation did not significantly increase the ratio of LC3 II/LC3 I compared with the ratio observed using irradiation alone (Fig. 1e), further demonstrating that only CpG ODN107 could increase autophagy.

#### CpG ODN107+ irradiation also increases the ratio of LC3 II/LC3 I in U251 and CHG-5 glioma cells

To investigate the presence of a radiosensitizing effect of CpG ODN107 for different glioma cell lines with different p53 and different PTEN statuses, U251 and CHG-5 glioma cell lines were used in this study. According to previous studies, PTEN and p53 genes are mutated in U251 cells; U87 is a cell line with the wild-type p53 gene^24^,^25^.

Our results showed that CHG-5 cells express PTEN but not p53 (Fig. S3). Therefore, U87, U251, and CHG-5 cells possessed different p53 and PTEN status. Subsequently, the radiation sensitization effect and mechanism of CpG ODN107 in U251 and CHG-5 cells were evaluated. The results showed that CpG ODN107+ irradiation significantly inhibited CHG-5 and U251 cell proliferation, and also increased the ratio of LC3 II/LC3 I compared with the results observed using irradiation alone (Fig. 1f,g), demonstrating that CpG ODN107 had a radiosensitizing effect on these two cell lines, which was closely related to autophagy despite the different p53 and different PTEN statuses.

#### 3-methyladenine (3-MA) decreases the radiosensitizing effect of CpG ODN107 and autophagy induced by CpG ODN107+ irradiation

3-MA is a well-known autophagy inhibitor^26^; thus, reversal of the effect induced by CpG ODN107 by 3-MA can further demonstrate that autophagy contributes to the radiosensitizing effect. Based on the above consideration, 4 mM of 3-MA was used in our experiments after this concentration of 3-MA showed no significant influence on U87 cell viability (data not shown).

**Figure 2:**
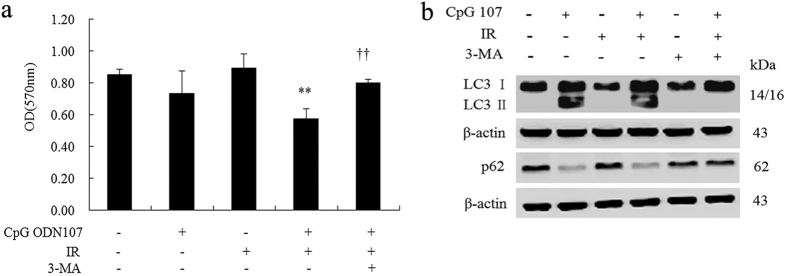
3-MA reverses the radiosensitizing effect of CpG ODN107 in U87 cells. (**a**) 3-MA reversed the radiosensitizing effect of CpG ODN107. Cells were treated with or without of 3-MA (4 mM) for 2 h, and then incubated with CpG ODN107 (10 μg/mL) for 12 h, before treatment with or without 5 Gy of irradiation. After incubation for another 24 h, the cells were collected for MTT assay. **P < 0.01 vs. IR; ^††^*P* < 0.01 vs. CpG 107 + IR. Error bars represent the mean ± S.D. (n = 5). **(b**) 3-MA reversed the autophagy-related proteins expressions. The cells were treated as described as (**a**), except the cells were collected for western blotting assay. In figures, CpG ODN107 was abbreviated as CpG 107; irradiation was abbreviated as IR.

The results of the MTT assay showed that CpG ODN107 possessed the radiosensitizing effect, but 3-MA reversed this effect (Fig. 2a). 3-MA alone could inhibit the expression of autophagy-related proteins (Fig. 2b), but more significantly, 3-MA could reverse the effect of CpG ODN107+ irradiation on the ratio of LC3 II/LC3 I and p62 protein expression (Fig. 2b). The above results further confirmed that autophagy is closely related to the radiosensitizing effect of CpG ODN107.

### CpG ODN107+ irradiation induces autophagy in a nude mouse orthotopic implantation tumor model

**Figure 3:**
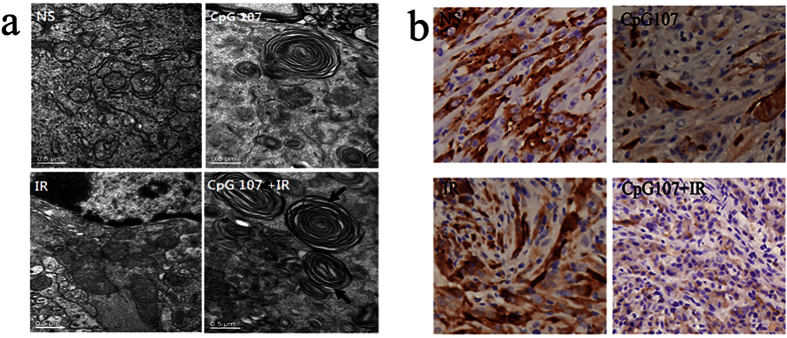
CpG ODN107 combined with local radiotherapy induces autophagosome formation of tumor cells in nude mice with orthotopic transplantation tumor. (**a**) The autophagosomes formation observed by TEM. Autophagosomes (marked by arrows) were presented as rounded vacuolar structures with double or multiple membranes surrounding the cytoplasmic contents. (**b**) Immunohistochemical analysis of p62 protein expression. p62 protein (brown) was expressed in the cytoplasm. In figures, CpG ODN107 was abbreviated as CpG 107; radiotherapy was abbreviated as IR.

Our previous results demonstrated that CpG ODN107 enhances the radiosensitivity of glioma cells to local radiotherapy in an orthotopic implantation tumor model^9^. In the current study, this model was used to investigate autophagy within tumors treated with CpG ODN107+ local radiotherapy *in vivo*. The double or multiple membrane structures typical of autophagosomes were not observed in mice treated with normal saline (NS) and radiotherapy alone. However, these structures were observed in mice treated with CpG ODN107 or CpG ODN107+ radiotherapy (Fig. 3a), confirming that the radiosensitizing effect of CpG ODN107 *in vivo* was also closely related to autophagy.

#### CpG ODN107+ local radiotherapy decreases p62 expression

The results of immunohistochemical analysis of the implanted tumor sections demonstrated that CpG ODN107+ local radiotherapy significantly decreased p62 expression compared with local radiotherapy alone (Fig. 3b), further demonstrating that the radiosensitizing effect of CpG ODN107 was closely related to the induction of autophagy in glioma not only *in vitro*, but also *in vivo.*

### The TLR9-ERK-mTOR signaling pathway is critical for autophagic cell death induced by CpG ODN107+ irradiation in glioma cells

#### CpG ODN107+ irradiation downregulates p-mTOR expression in U87 cells

**Figure 4:**
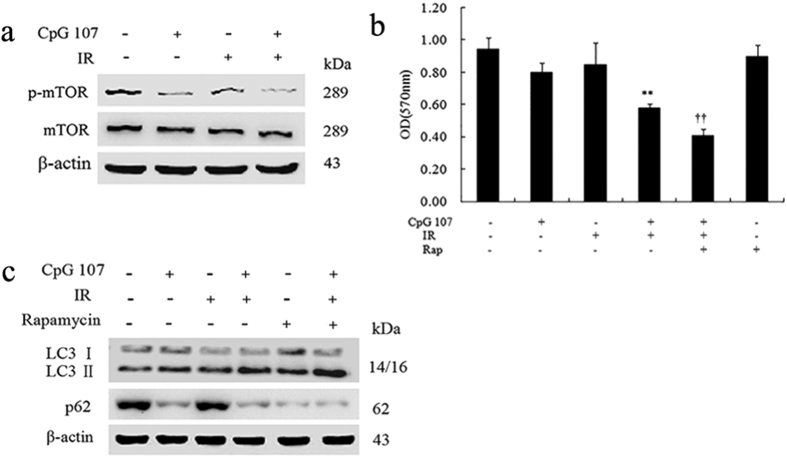
CpG ODN107+ irradiation affects autophagy-ralated proteins expression in U87 cells. (**a**) p-mTOR and mTOR protein expression in U87 cells treated with CpG ODN107+ irradiation. Cells were treated with 10 μg/mL of CpG ODN107 for 12 h, and then treated with or without 5 Gy of irradiation. After incubation for another 24 h, cells were collected for western blotting assay. (**b**) The effects of rapamycin on the cell viability. Cells were pretreated with rapamycin (10 nM) for 1 h, and then incubated with CpG ODN107 (10 μg**/**mL) for 12 h with or without 5 Gy of irradiation. After incubation for another 24 h, the cells were collected for MTT assay. **P < 0.01 vs. IR; ^††^*P *< 0.01 vs. CpG ODN107+ IR. Error bars represent the mean ± S.D. (n = 5). (**c**) The effects of rapamycin on LC3 and p62 proteins expressions. The cells were treated as described as (**b**), except the cells were collected for western blotting assay. In figures, CpG ODN107 was abbreviated as CpG 107; irradiation was abbreviated as IR; Rapamycin was abbreviated as Rap.

mTOR is one of the main negative regulators of autophagy^27^; therefore, the ability of CpG ODN107+ irradiation to inhibit the phosphorylation of mTOR protein (p-mTOR) was investigated. As expected, CpG ODN107+ irradiation caused a significant reduction in p-mTOR expression compared with the effect when irradiation alone was used (Fig. 4a), suggesting that mTOR was involved in CpG ODN107-induced autophagy in U87 cells.

Subsequently, rapamycin, the mTOR inhibitor, was used to investigate whether p-mTOR is an essential molecule for autophagy induced by CpG ODN107+ irradiation. The results showed that rapamycin indeed increased the radiosensitizing effect of CpG ODN107 (Fig. 4b) and the ratio of LC3 II/LC3 I (Fig. 4c) in cells treated with CpG ODN107+ irradiation, demonstrating that autophagy induced by CpG ODN107+ irradiation is closely related to mTOR signaling.

#### CpG ODN107+ irradiation upregulates p-ERK in U87 cells

**Figure 5:**
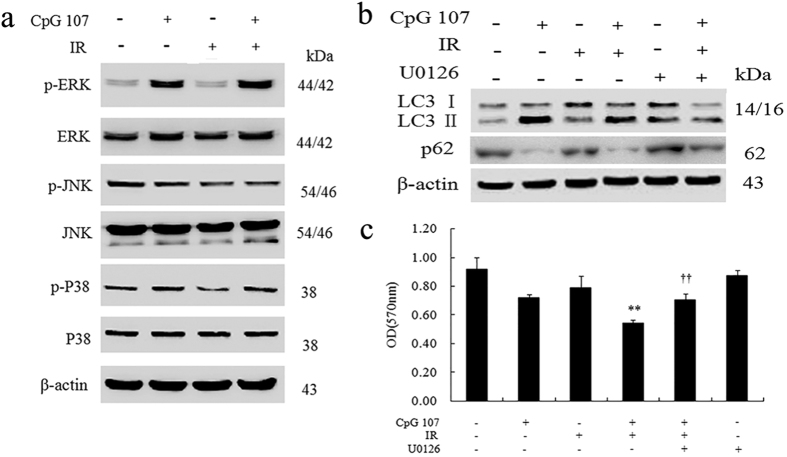
CpG ODN107+ irradiation has different influences on MAPKs in U87 cells. (**a**) ERK, JNK, P38 proteins expressions in U87 cells. Cells were treated with 10 μg/mL of CpG ODN107 for 12 h, and then treated with or without 5 Gy of irradiation. After incubation for another 24 h, the cells were collected for western blotting assay. (**b**) The effects of U0126, an inhibitor of ERK, on LC3 and p62 protein expression. Cells were pretreated with U0126 (10 μM) for 2 h, and then incubated with CpG ODN107 (10 μg/mL) for 12 h with or without 5 Gy of irradiation. After incubation for another 24 h, the cells were collected for western blotting assay. (**c**) The effect of U0126 on the cell viability. The cells were treated as described as (**b**), except the cells were collected for MTT assay. Error bars represent the mean ± S.D. (n = 5). **P < 0.01 vs. IR; ^††^*P *< 0.01 vs. CpG ODN107 + IR. In figures, CpG ODN107 was abbreviated as CpG 107; irradiation was abbreviated as IR.

Several signaling pathways such as the MAPK pathway have been reported to regulate autophagy via mTOR pathway^28^,^29^,^30^,^31^. To determine the role of different pathways in autophagy induced by CpG ODN107+ irradiation, JNK, ERK, and p38 proteins and the expressions of their phosphorylated forms were investigated. The results showed only p-ERK level not total ERK level increased obviously in cells treated with CpG ODN107+ irradiation compared with irradiation alone. However, there were no change of their phosphorylated forms and the total levels of p38 and JNK proteins (Fig. 5a).

Therefore, in subsequent experiments, an ERK inhibitor (U0126) was used to further confirm the role of ERK in autophagy induced by CpG ODN107+ irradiation. The results showed that U0126 significantly decreased the ratio of LC3 II/LC3 I (Fig. 5b) and reversed the radiosensitizing effect of CpG ODN107 (Fig. 5c), demonstrating that ERK was important for autophagy induced by CpG ODN107+ irradiation and its radiosensitizing effect of CpG ODN107.

#### CpG ODN107+ irradiation upregulates TLR9 expression in U87 cells

TLR9, the key pattern recognition receptor for CpG ODN, activates the MyD88-dependent pathway, leading to the activation of MAPKs and NF-κB. In this study, the role of TLR9 was investigated to determine whether autophagy induced by CpG ODN107+ irradiation was mediated via the TLR9 signaling pathway.

**Figure 6:**
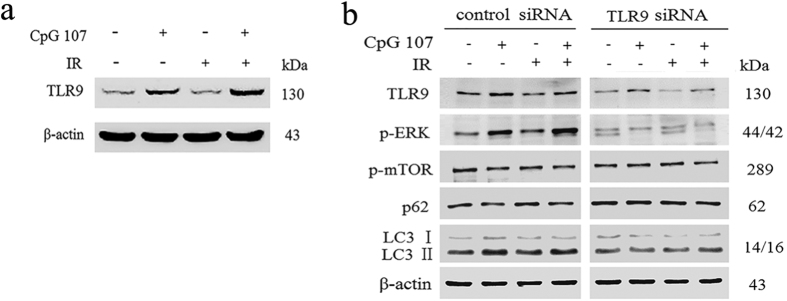
CpG ODN107+ irradiation has different influences on important signaling molecules in U87 cells treated with or without TLR9 siRNA. (**a**) TLR9 protein expression. Cells were treated with 10 μg/mL of CpG ODN107 for 12 h, and then treated with or without 5 Gy of irradiation. After incubation for another 24 h, the cells were collected for western blotting assay. (**b**) The effect of TLR9 siRNA on TLR9, p-ERK, p-mTOR, LC3 and p62 proteins expressions. Cells were transfected with control siRNA or TLR9-specific siRNA. All transfection was performed for 24 h prior to treatment with 10 μg/mL of CpG ODN107 combined with 5 Gy of irradiation. After incubation for another 24 h, the cells were collected for western blotting assay. In figures, CpG ODN107 was abbreviated as CpG 107; irradiation was abbreviated as IR.

The results of western blotting showed that CpG ODN107+ irradiation could significantly increase TLR9 protein expression than irradiation alone (Fig. 6a). Subsequently, siRNA technology was employed to further determine the role of TLR9. The results showed that CpG ODN107+ irradiation increased neither the expression of neither TLR9 nor p-ERK nor the ratio of LC3 II/LC3 I, and didn’t decrease the expression of p-mTOR and p62 within U87 cells treated with TLR9-specific siRNA. In contrast, control siRNA had no influence on above proteins (Fig. 6b). The results demonstrated that TLR9 indeed played a key role in autophagy induced by CpG ODN107+ irradiation.

#### CpG ODN107+ irradiation upregulates TLR9 and p-ERK expression, and downregulates p-mTOR expression in CHG-5 and U251 cells

**Figure 7:**
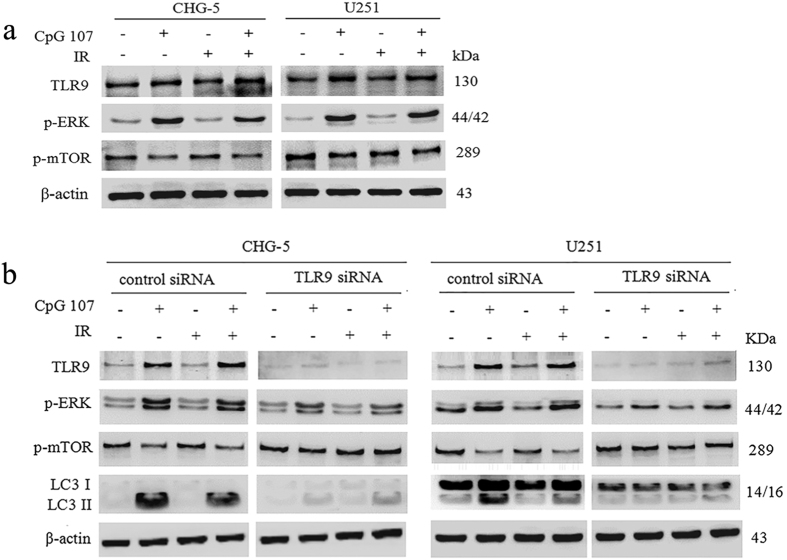
CpG ODN107 + irradiation upregulates TLR9, p-ERK expression and downregulates p-mTOR expression in CHG-5 and U251 cells. (**a**) Cells were treated with 10 μg/mL of CpG ODN107 for 12 h, and then treated with or without 5 Gy of irradiation. After incubation for another 24 h, the cells were collected for western blotting assay. (**b**) The effect of TLR9 siRNA on TLR9, p-ERK, p-mTOR and LC3 proteins expressions. Cells were transfected with control siRNA or TLR9-specific siRNA. All of transfection was performed for 24 h prior to treatment with 10 μg/mL of CpG ODN107 combined with 5 Gy of irradiation. After incubation for another 24 h, the cells were collected for western blotting assay. In figures, CpG ODN107 was abbreviated as CpG 107; irradiation was abbreviated as IR.

The expression of TLR9, p-ERK, and p-mTOR was also investigated to confirm the effects observed in CHG-5 and U251 cells. The results showed that CpG ODN107+ irradiation increased TLR9 and p-ERK expressions but decreased p-mTOR expression compared with the results when irradiation alone was used (Fig. 7a). Subsequently, siRNA technology was employed to further determine the role of TLR9 in CHG-5 and U251 cells. The results also showed that CpG ODN107+ irradiation increased neither the expression of neither TLR9 nor p-ERK nor the ratio of LC3 II/LC3 I, and did not decrease the expression of p-mTOR and p62 within CHG-5 and U251 cells treated with TLR9-specific siRNA (Fig. 7b). These observations in CHG-5 and U251 cells were in line with the results observed in U87 cells.

## Discussion

The results of the present study demonstrated that the radiosensitizing effect of CpG ODN107 was closely related to autophagic cell death, and that TLR9 played a key role during autophagy. Further, the TLR9-ERK-mTOR signaling pathway was critical for this type of autophagic cell death induced by CpG ODN107+ irradiation in glioma cells.

Autophagy, also known as type II programmed cell death, is originally described as a process of protein degradation and recycling in response to starvation or stress. It is well known that irradiation could induce autophagy^17^. Therefore, in our present experiments, the effects of different irradiation doses on autophagosome formation and autophagy-related protein expression were investigated. After U87 cells were irradiated with 0, 3, 5, 7, and 9 Gy of irradiation for 24 h, TEM observation showed that autophagosome could be found within U87 cells treated with any of the irradiation dosage; however, western blotting showed only that irradiation dosages of 7 Gy and 9 Gy for 24 h could significantly increase the ratio of LC3 II/LC3 I in U87 cells compared with the cells in the no irradiation group. Based on previous results that U87 cells were insensitive to 5 Gy irradiation and that 10 μg/mL of CpG ODN107 combined with 5 Gy of irradiation could significantly inhibit the proliferation and viability of U87 cells^8^,^9^, the dose of 5 Gy of irradiation, insensitive for glioma cells, and 10 μg/mL of CpG ODN107 was selected and used in our present experiments.

In some tumor cells, autophagy can be a protective response enabling survival against anticancer treatments through blockade of the apoptotic pathway. Meanwhile, other tumor cells undergo autophagic cell death in response to cancer therapies^32^. Although radiosensitizing treatment or radiotherapy can induce apoptosis in malignancies such as small-cell lung cancer, nasopharyngeal carcinoma cells, and colon cancer cells^33^,^34^,^35^,^36^, several reports demonstrate that autophagy rather than apoptosis significantly contributes to the antitumor effects of radiosensitizing treatment or radiotherapy in glioma. Interestingly, this phenomenon is usually observed in cells resistant to apoptotic cell death. Arsenic trioxide treatment combined with irradiation enhanced the autophagic effects in U118-MG cells that were resistant to various pro-apoptotic therapies^37^,^38^,^39^,^40^,^41^.

CpG ODN is considered as a promising radiosensitizer^6^,^7^. This type of molecule was capable of exerting its radiosensitizing effect via inducing apoptosis and cell cycle arrest^42^,^43^. Recently, CpG ODN was found to inhibit tumor cell growth via inducing autophagy in TLR9-positive tumor cells of colon carcinoma^18^,^44^. However, its radiosensitive effect against tumor cells has not been confirmed to be mediated via autophagic cell death. Previously, in our lab, CpG ODN107 was demonstrated to exert a radiosensitizing effect both *in vitro* and *in vivo*; interestingly, this effect was not related to apoptosis as well^8^,^9^. In this study, we demonstrated that CpG ODN107 exerted its radiosensitizing effect via inducing autophagic cell death in glioma cells not only *in vitro* but also *in vivo*, confirming that the autophagic cell death of tumor cells was closely related to the radiosensitizing effect, in line with the results from other labs.

Because more drugs that potentially modulate autophagy are increasingly being used in clinical trials for therapeutic purposes, it is important to determine whether these drugs are truly affecting autophagy^23^. Morphologically, autophagy is characterized by the formation of a double-layered isolation membrane. Therefore, TEM observation is the gold standard method for determination of autophagy^19^. Additionally, several molecules are involved in the process of autophagosome formation; essential autophagy-related proteins such as LC3 (light chain 3) and p62 are the important key markers^20^. Our results demonstrated that CpG ODN107+ irradiation could induce autophagosome formation within U87 cells and especially increase beclin 1, the ratio of LC3 II/ LC3 I, decrease p62 protein expression, and induce punctate GFP-LC3 formation within U87 cells with stable GFP-LC3 expression. However, non-CpG ODN had no such effect. Therefore, our results further demonstrated that autophagy was closely related to the radiosensitizing effect of CpG ODN107+ irradiation.

U87 was one of the most used malignant glioma cell line in the present study, since this cell line has a unique feature of wild-type p53. Since extensive evidences show that p53 is related to autophagy response, different cell lines with different P53 and PTEN statuses such as U251 cell and CHG-5 were investigated. Our results showed that CpG ODN107+ irradiation also significantly inhibited CHG-5 and U251 cell proliferation and increased the ratio of LC3 II/LC3 I compared with when irradiation alone was used, suggesting that CpG ODN107+ irradiation had a radiosensitizing effect on different malignant glioma cell lines, and this radiosensitizing effect was related to autophagy response as well.

Autophagy is a complicated regulatory process regulated by several upstream signaling pathways. The mammalian target of rapamycin (mTOR) serves as the main regulator of autophagy in both normal and cancer cells; mTOR activation suppresses autophagy and stimulates cell proliferation in response to nutrient availability. Under conditions of nutrient deprivation or other stresses, the suppression of mTOR stimulates the autophagic cascade and inhibits cell proliferation^45^,^46^, but the activation of PI3K-AKT-mTOR, AMPK-mTOR, MAPK-mTOR, p53, beclin 1, and the Bcl-2 pathway stimulates autophagy. Our results demonstrated that CpG ODN107+ irradiation repressed mTOR phosphorylation. Further, the mTOR inhibitor rapamycin increased the induction of autophagy and further increased the radiosensitizing effect of CpG ODN107. These data demonstrated that the mTOR signaling pathway was closely involved in autophagy induced by CpG ODN107+ irradiation and contributed to the radiosensitizing effect of CpG ODN107.

CpG ODN is a known regulator of innate and acquired immunity. Typically, CpG ODN is first internalized by cells and then recognized by TLR9 in the early endosome, leading to activation of the MyD88-dependent pathway, NF-κB, and MAPKs including c-Jun N-terminal kinase (JNK), p38 and ERK, which then induce the release of nitrous oxide and pro-inflammatory cytokines^47^,^48^. CpG ODN has been found to induce autophagy in tumor cell lines such as colon and prostate cancers in a TLR9-dependent manner^18^,^44^. However, the exact signaling pathways involved in autophagy induced by CpG ODN+ irradiation remain unclear. In this study, based on the recognition of the crosstalk between the TLR9-MAPK signaling pathway in innate immunity and the MAPK-mTOR signaling pathway in autophagy, we focused on the role of TLR9-MAPK in the possible mechanism of autophagy in U87, CHG-5 and U251 cells. Our findings revealed that CpG ODN107+ irradiation significantly induced TLR9 and p-ERK protein upregulation. The ERK inhibitor reversed the radiosensitizing effect of CpG ODN107 in glioma cells and inhibited the autophagy induced by CpG ODN107+ irradiation, demonstrating that ERK-mediated signaling pathway was related to the autophagy. Further, on treating U87, CHG-5 and U251 cells with TLR9-specific siRNA, the results showed that CpG ODN107+ irradiation did not increase p-ERK expression and the ratio of LC3 II/LC3 I, and did not decreased the expressions of p-mTOR and p62 proteins. These results further demonstrated that TLR9 played a key role and that ERK rather than p38 and JNK was involved in autophagy induced by CpG ODN107+ irradiation and in the radiosensitizing effect of CpG ODN107. Therefore, our research established a molecular connection among TLR9, ERK, and mTOR in CpG ODN107-induced autophagy, providing an improved understanding of the signaling pathway involved in autophagy during radiosensitization.

**Figure 8:**
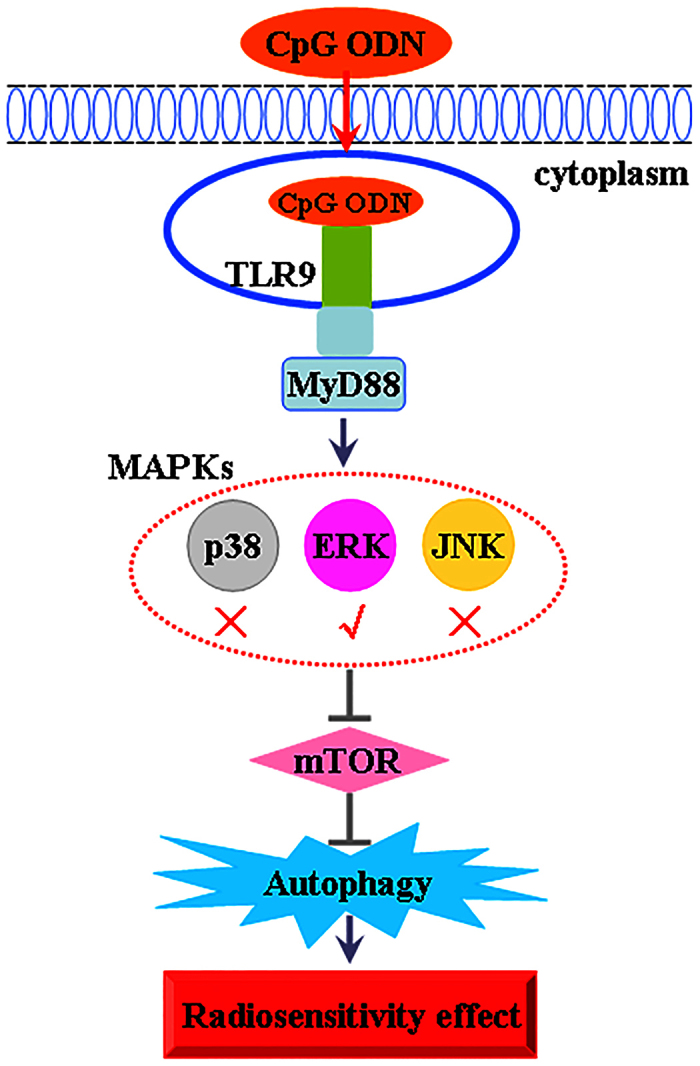
A schematic diagram of autophagy induced by CpG ODN107 combined with irradiation in glioma cells.

In conclusion, we demonstrated that CpG ODN107+ irradiation induces autophagic cell death via the TLR9-ERK-mTOR signaling pathway in glioma cells (Fig. 8), thus, further elucidating the mechanism by which CpG ODN107+ irradiation induces autophagy and providing new insights into the radiosensitizing effects of CpG ODN107 in tumor cells. These findings might be applicable to the development of novel cancer treatments and more generally, for gene therapy approaches in TLR9-positive tissues.

## Materials and Methods

### Reagents

Methyl-thiazdyldiphenyl-tetrazolium bromide (MTT), dimethyl sulfoxide (DMSO), 3-MA, rapamycin and the ERK inhibitor (U0126) were purchased from Sigma (NY, USA). LC3, beclin 1, TLR9, (p)-mTOR, (p)-JNK, (p)-ERK and (p)-p38 monoclonal antibodies were purchased from Cell Signaling Technology (Beverly, MA, USA). TLR9 siRNA was purchased from Santa Cruz Biotechnology (Santa Cruz, CA, USA). Cell Lysis Buffer, BCA Protein Assay Kits and BeyoECL plus Kits were purchased from Beyotime (Shanghai, China). The Lipofectamine^TM^ 2000 transfection reagent was purchased from Invitrogen (Shanghai, China). The CpG ODN107 (5′-TGGCGCGGGGCG-3′) and Non-CpG ODN (a conversed CG sequence, 5′ -TCCATGAGCTTCCTGATGCT- 3′) were used in the experiments incorporated a phosphorothioate backbone and was synthesized by Invitrogen Ltd. Co. (Shanghai, China). It was dissolved and diluted in phosphate-buffered saline (0.01 M PBS, pH 7.4) for *in vitro* experiments, and dissolved and diluted in normal saline (NS, 0.9%) for *in vivo* experiments.

### Cell culture

Human glioma U87 (glioblastoma multiform, WHO IV) and U251 cell line (glioblastoma multiform, WHO IV) were purchased from the American Type Culture Collection. Human glioma CHG-5 cell line (glioblastoma multiform, WHO grade II, very commonly used in China) was kindly provided by Prof. Xiuwu Bian (Southwestern Hospital, Chongqing, China). The cells were cultured in Dulbecco’s modified Eagle’s medium (DMEM) supplemented with 10% fetal bovine serum (Gibco, USA) and antibiotics (100 U/ml penicillin and 100 μg/mL streptomycin) in a 5% CO_2_ atmosphere at 37 °C. Endotoxin levels in cell culture media and supernatants were undetectable (<1 ng/mg) as assessed by Limulus assay.

### Irradiation

*In vitro*, U87 cells were exposed to β-ray irradiation at a single dose of 5 Gy using 2300 C/D accelerator linear (Varian, Chicago, USA). In orthotopic nude mice model, local radiotherapy was carried out at a single dose of β-ray irradiation (10 Gy, 7 MeV) using 2 300 C/D accelerator linear, other areas of the mouse body were protected by a grid. For radiotherapy, the nude mice were anesthetized with pentobarbital sodium (35 mg/kg, Sigma, Steinheim, Germany) via intraperitoneal injection. The dose used for *in vitro* experiments was lower than that used for *in vivo* experiments because well-distributed growing cells in culture plates are more susceptible to irradiation.

### The orthotopic implantation model and treatment

The method used to establish the orthotopic implantation model and the treatment of tumor-bearing nude mice was in accordance with a previous study^9^. Tumor-bearing nude mice were randomly divided into four groups (3 mice/group): Group 1 received an intratumoral injection of NS (5 μL), Group 2 received an intratumoral injection of 0.083 mg/kg of CpG ODN107 (5 μL), Group 3 received local radiotherapy, and Group 4 received an intratumoral injection of CpG ODN107 (0.083 mg/kg) in combination with a single dose of local radiotherapy. Mice were anesthetized on Day 30 after treatment. The brains were then collected and fixed with 4% paraformaldehyde, embedded in paraffin, and sectioned for immunohistochemistry assay.

### MTT assay

Cells (1.0 × 10^4^/mL) were seeded in 96-well plates and pretreated on the following day with 4 mM of 3-MA for 1 h, 10 nM of rapamycin for 1 h or 10 μM of U0126 for 2 h. Cells were then treated with 10 μg/mL of CpG ODN107 for 12 h prior to being treated with or without irradiation. After incubation for a further 24 h, MTT assays were carried out using a standard protocol and optical density (OD) was read at 570 nm using the ELISA analyzer (Bio-Rad).

### Colony formation assay

Cells (200 cells/dish) were seeded onto 60-mm dishes in three independent experiments and treated with 10 μg/mL of CpG ODN107 or vehicle for 12 h. They were then treated with or without 5 Gy of irradiation. After culturing for 10 days, the surviving colonies were stained with Giemsa stain. Colonies with more than 50 cells were counted under an inverted microscope. The survival fraction (%) was calculated according to the following formula: colony number of the treated group/colony number of the medium group ×100%.

### Transmission electron microscopy (TEM)

Cells (5.0 × 10^4^/mL) were seeded in cell culture bottles before being treated on the following day with 10 μg/mL of CpG ODN107 for 12 h, followed by treatment with or without irradiation. After incubation for a further 24 h, cells were collected and fixed in cold 2.5% glutaraldehyde in phosphate buffered saline (PBS). The specimens were post-fixed in 1% osmium tetroxide with 0.1% potassium ferricyanide, dehydrated through a graded series of ethanol (30–90%), and embedded in Epon. Ultrathin sections (65 nm) were stained with 2% uranyl acetate and Reynold’s lead citrate, and imaged using a JEOL JEM-1011 TEM at 80 KV. Images were captured using a side-mount AMT 2k digital camera (Advanced Microscopy Techniques, Danvers, MA, USA).

### Transfection and establishment of a stable GFP-LC3 expressing U87 cell line

The GFP-LC3 plasmid was transfected into U87 cells using Lipofectamine^TM^ 2000 (Invitrogen, Shanghai, China). GFP-LC3-expressing U87 cells were then sorted by flow cytometry (Becton Dickinson, US). The stable transfectants were maintained in 300 mg/mL of G418.

### Laser confocal scanning

GFP-LC3-expressing U87 cells (2.0 × 10^5^/mL) were seeded into a confocal dish (35 mm diameter). Cells were treated on the following day with CpG ODN107 (10 μg/mL) or vehicle for 12 h, followed by treatment with or without irradiation. After incubation for a further 24 h, the cells were washed three times with warm PBS and fixed in 4% paraformaldehyde for 15 min at room temperature. The fixed cells were washed three times in PBS. Nuclear DNA was stained with diamidino-phenyl-indole (DAPI, blue) for 6 min. Cells were then washed three times with PBS and examined using a laser confocal scanning microscope (Leica TCS-NT, Mannheim, Germany).

### Western blotting

Cells (5.0 × 10^4^/mL) were seeded in cell culture bottles before being treated on the following day with 10 μg/mL of CpG ODN107 for 12 h, followed by treatment with or without irradiation. After incubation for a further 24 h, cells were collected. Total cell protein was extracted with Cell Lysis Buffer and the protein concentration was measured with a BCA Protein Assay Kit (Beyotime Institute of Biotechnology, Beijing, China). Subsequently, the proteins were separated by SDS-polyacrylamide gel electrophoresis (SDS-PAGE) and transferred to PVDF membranes (EMD Millipore, Boston, MA, USA). After blocking with dried skimmed milk in TBST (10 mM Tris-HCl, 0.1 M NaCl_2_, 0.1% Tween 20, pH 7.4), membranes were incubated with specific primary antibodies (1:1,000) prior to incubation with HRP-conjugated secondary antibodies (1:10,000). Proteins were visualized using the BeyoECL plus Kit (Beyotime Institute of Biotechnology, Beijing, China). Immunoblotting signals were scanned and quantitatively determined using a ChemiDoc XRS gel imaging system (Bio-Rad, Hercules, CA, USA) and Quantity One software, respectively.

### Immunohistochemistry staining and immunofluorescence assay

The brain sections were dewaxed, soaked in ethanol, and then blocked with 3% H_2_O_2_. Non-specific immunoreactivity was blocked with diluted normal rabbit serum at room temperature. The sections were then incubated overnight at 4 °C with anti-LC3 antibody, anti-p62 antibody, anti-TLR9 antibody, anti-pERK or anti-pmTOR antibody, which was diluted in blocking buffer. The sections were further incubated with a biotinylated secondary antibody (diluted 1:500), stained with freshly prepared diaminobenzidine solution, and then counterstained with Mayer’s hematoxylin. After each step, the sections were washed with PBS. The negative control was obtained by substituting the primary antibodies with mouse immunoglobulin G. For immunofluorescence assay, the sections were treated with the primary antibodies as described and then incubated with a secondary antibody conjugated with red or green fluorescence. Finally, the sections were mounted and examined under a laser confocal scanning microscope (Leica TCS-NT).

### Autophagy assays

Autophagy was evaluated in cells by laser confocal scanning microscopy, TEM or western blotting. In laser confocal scanning microscopy experiments, the percentage of GFP-LC3 positive cells expressing GFP-LC3 punctate dots (type II of the autophagy marker LC3) was counted. In TEM experiments, autophagy was evaluated by observing the typical double membrane vesicles^19^. In western blotting experiments, beclin 1, the conversion of LC3 I to LC3 II, and p62 protein expression (the marker protein of autophagy) were detected.

### Statistical analysis

All results were presented as mean ± standard deviation. Data were analyzed by Student’s *t*-test or ANOVA, and *p*-values less than 0.05 were considered to indicate statistically significant differences.

## Additional Information

**How to cite this article**: Li, X. *et al*. TLR9-ERK-mTOR signaling is critical for autophagic cell death induced by CpG oligodeoxynucleotide 107 combined with irradiation in glioma cells. *Sci. Rep.* **6**, 27104; doi: 10.1038/srep27104 (2016).

## Supplementary Material

Supplementary Information
