# Supplementary material for: TLR9-ERK-mTOR signaling is critical for autophagic cell death induced by CpG oligodeoxynucleotide 107 combined with irradiation in glioma cells
Source: Sci Rep. 2016 Jun 2;6:27104. doi: 10.1038/srep27104 (PMC4890034; doi:10.1038/srep27104)
Supplement: Supplementary Information [file srep27104-s1.doc]

**TLR9-ERK-mTOR signaling is critical for autophagic cell death induced by CpG oligodeoxynucleotlde 107 combined with irradiation in glioma cells**

Xiaoli Li ^a^, Yanyan Cen ^a^, Yongqing Cai ^b^, Tao Liu ^a^, Huan Liu ^a^, Guanqun Cao ^a^, Dan Liu ^a^, Bin Li ^a^, Wei Peng ^a^, Jintao Zhou ^c^, Xueli Pang ^d^, Jiang Zheng ^e, *^, Hong Zhou ^a, *^


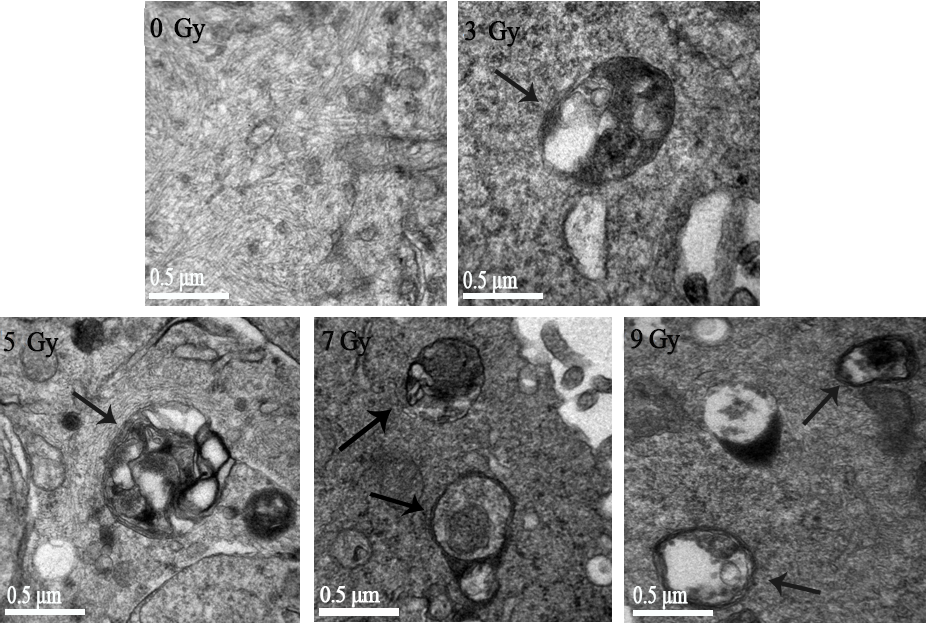


**Figure S1** Autophagosomes formation in U87 cells exposed to different doses of irradiation. Cells were treated with different doses of irradiation for 24 h, and then were collected for transmission electron microscopy (TEM) observation. Autophagosomes (marked by arrows) were presented as rounded vacuolar structures with double or multiple membranes surrounding the cytoplasmic contents.


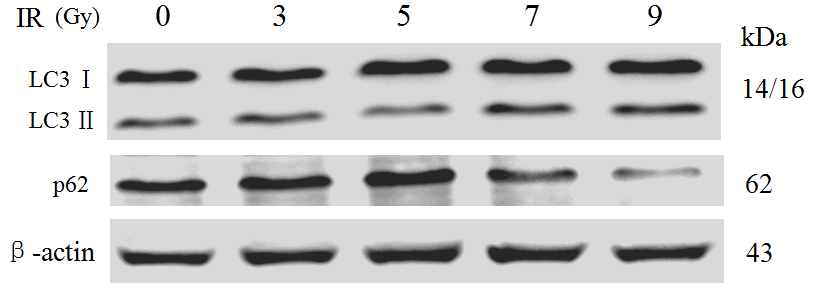


**Figure S2** LC3 and p62 protein expressions in U87 cells exposed to different doses of irradiation. Cells were treated with different doses of irradiation for 24 h, and then cells were collected for western blotting assay.


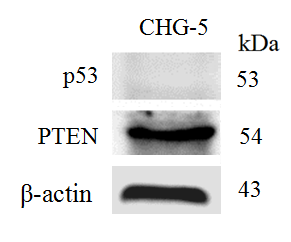


**Figure S3** p53 and PTEN proteins expressions in CHG-5 cells. Cells were seeded in 6-well plates and incubated for 24 h, and then were collected for western blot assay.
